# Profile of Nagasaki Islands Study (NaIS): A Population-based Prospective Cohort Study on Multi-disease

**DOI:** 10.2188/jea.JE20230079

**Published:** 2024-05-05

**Authors:** Jun Miyata, Hirotomo Yamanashi, Shin-Ya Kawashiri, Sakiko Soutome, Kazuhiko Arima, Mami Tamai, Fumiaki Nonaka, Yukiko Honda, Masayasu Kitamura, Koji Yoshida, Yuji Shimizu, Naomi Hayashida, Shigeru Kawakami, Noboru Takamura, Takashi Sawase, Atsutoshi Yoshimura, Yasuhiro Nagata, Mayumi Ohnishi, Kiyoshi Aoyagi, Atsushi Kawakami, Toshiyuki Saito, Takahiro Maeda

**Affiliations:** 1 Department of Island and Community Medicine, Nagasaki University Graduate School of Biomedical Sciences, Nagasaki, Japan; 2 Department of General Medicine, Nagasaki University Graduate School of Biomedical Sciences, Nagasaki, Japan; 3 Department of Community Medicine, Nagasaki University Graduate School of Biomedical Sciences, Nagasaki, Japan; 4 Department of Oral Health, Nagasaki University Graduate School of Biomedical Sciences, Nagasaki, Japan; 5 Department of Public Health, Nagasaki University Graduate School of Biomedical Sciences, Nagasaki, Japan; 6 Department of Immunology and Rheumatology, Nagasaki University Graduate School of Biomedical Sciences, Nagasaki, Japan; 7 Center for Advanced Preventive Medical Sciences, Nagasaki University Graduate School of Biomedical Sciences, Nagasaki, Japan; 8 Department of Health Sciences, Nagasaki University Graduate School of Biomedical Sciences, Nagasaki, Japan; 9 Department of Public Health, Osaka Institute of Public Health, Osaka, Japan; 10 Division of Strategic Collaborative Research, Atomic Bomb Disease Institute, Nagasaki University, Nagasaki, Japan; 11 Department of Pharmaceutical Informatics, Nagasaki University Graduate School of Biomedical Sciences, Nagasaki, Japan; 12 Department of Global Health, Medicine and Welfare, Atomic Bomb Disease Institute, Nagasaki University, Nagasaki, Japan; 13 Department of Applied Prosthodontics, Nagasaki University Graduate School of Biomedical Sciences, Nagasaki, Japan; 14 Department of Periodontology and Endodontology, Nagasaki University Graduate School of Biomedical Sciences, Nagasaki, Japan; 15 Department of Public Health Nursing, Nagasaki University Graduate School of Biomedical Sciences, Nagasaki, Japan; 16 Leading Medical Research Core Unit, Nagasaki University Graduate School of Biomedical Sciences, Nagasaki, Japan

**Keywords:** aging, cohort studies, Japanese, noncommunicable diseases, risk factors

## Abstract

In an aging society, it is important to visualize the conditions of people living with diseases or disabilities, such as frailty and sarcopenia, and determine the environmental and genetic factors underlying such conditions. Atherosclerosis and arterial stiffness are key conditions between these factors and noncommunicable diseases. In 2014, we launched a population-based prospective open-cohort study, the Nagasaki Islands Study (NaIS), which was conducted in Goto City, located in the remote islands of Nagasaki Prefecture, Japan, mostly involving middle-aged and older residents. We conducted our own health checkups along with the annual standardized checkups organized by the municipality; recruited study participants; and started to follow them for vital status (death), migration, and occurrence of diseases, such as myocardial infarction, stroke, fracture, and human T-cell leukemia virus type 1 (HTLV-1)-associated uveitis. Our checkups were conducted as baseline surveys in different areas of Goto City during the fiscal years 2014–2016, secondary surveys during 2017–2019, and tertiary surveys since 2021, consisting of medical interviews, physical examinations, blood and urine tests, body composition measurements, osteoporosis screening, arterial stiffness measurements, carotid ultrasonography, and dental examination. A total of 4,957 residents participated in either the baseline or secondary surveys and were followed; 3,594 and 3,364 residents (aged 27–96 and 28–98 years) participated in the baseline and secondary surveys, respectively. In conclusion, the NaIS has been undertaken to reveal the influence of aging and risk factors of noncommunicable diseases and disabilities, with an aim to contribute towards better healthcare in the future.

## PURPOSE

The number of people living with diseases is increasing worldwide. From 2005 to 2013, the Global Burden of Disease (GBD) Study reported that the number of disability-adjusted life-years increased for most specific noncommunicable diseases, including cardiovascular diseases; accordingly, the years lived with disability (YLDs) also increased.^1^ The increase in life expectancy was greater than that in health-adjusted life expectancy (HALE) in most country-specific estimates.^1^ In Japan, there is a 9- and 11-year difference for men and women, respectively, between average life expectancy and HALE.^1^

Therefore, it is important to visualize the condition of people living with diseases or disabilities in an aging society. Recently, the concepts of multimorbidity and frailty have attracted attention,^2^ wherein fracture is also another important issue. The GBD study estimated 22 million YLDs from fractures in 2013,^3^ most of which caused long-term disabilities. Japan is one of the countries with the most advanced aging populations, and aging is even more relevant in remote islands. In 2010, the proportion of residents aged ≥65 years has already exceeded 30% in many remote islands.^4^ Furthermore, in 2010, the Japanese government stated that scientific research in life sciences, informatics, and genomics in pursuit of innovations should be promoted to improve diagnosis and treatment of disorders that affect aging societies.^5^ Studies in line with that concept were warranted.

Additionally, it is essential to clarify the environmental and genetic factors that underlie disease prevention. Atherosclerosis and arterial stiffness are key conditions that may mediate the link between environmental and genetic risk factors and the onset of cardiovascular diseases,^6^ which is a major burden.^1^ Recent reports have highlighted associations among vascular conditions and rheumatoid arthritis,^7^ other autoimmune diseases,^8^^,^^9^ and periodontitis.^10^ However, the underlying mechanisms have not been fully elucidated.

As a part of community-based prevention programs, Nagasaki University conducted many field surveys addressing health issues in remote islands. For example, we discovered that residents living in the non-main islands in Goto City had fewer teeth than residents of the main islands, owing to the lack of medical resources.^11^ We are addressing this issue by initiatives, such as establishing temporary dental clinics with the cooperation of the local government. Additionally, to prevent duplication and polypharmacy, we established a drug dispensing database to share prescription information with patients using multiple medical facilities and pharmacies; this was done with the support from Goto City and Goto Pharmaceutical Association.^12^ The data collected from all Goto City pharmacies could be used in our research. Our research demonstrates the potential for utilizing various real-world data with support from the local government.

Moreover, the prevalence of human T-cell leukemia virus type 1 (HTLV-1) is a major health concern in our field, particularly in Southwestern Japan and Nagasaki Prefecture, where the virus is highly endemic.^13^ Prior studies have suggested that HTLV-1 carriers are at an increased risk of death and various diseases.^14^ However, the mechanisms by which HTLV-1 exerts its adverse effects on carriers remain unclarified. This cohort study, whose target population had a higher likelihood of HTLV-1 carriers than the other cohort studies, could serve as a valuable reference for gaining insight into HTLV-1-associated diseases.

In 2014, we launched a population-based prospective open cohort study in remote islands, called the Nagasaki Islands Study (NaIS), representing the future of the aging population in Japan. This study aimed to identify the influence of aging on health conditions; elucidate the risk factors of cardiovascular diseases, rheumatic and autoimmune diseases, dental diseases, frailty, sarcopenia, osteoporosis, and fracture; and contribute to preventive medicine.

## MAIN FEATURES

**Figure 1.**
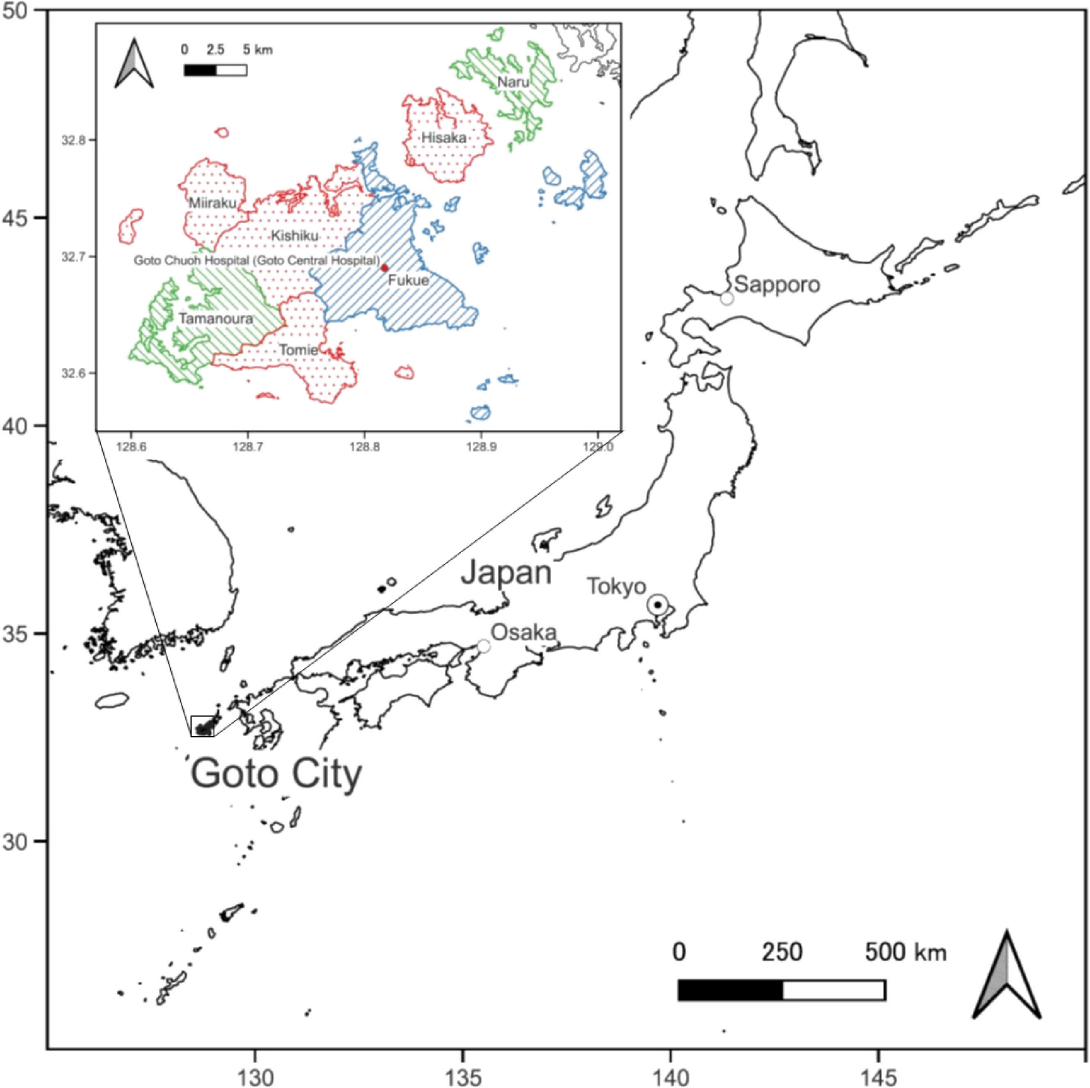
Map of the Nagasaki Islands Study (NaIS). We performed surveys at Fukue in the fiscal year 2014, 2017, 2021, and 2022 (area with blue stripe pattern); Tamanoura and Naru in 2015, 2018, and 2023 (green stripe pattern); Kishiku in 2016, 2019, and 2023 (red dot pattern); and Tomie, Miiraku, and Hisaka in 2016 and 2019 (red dot pattern).

The NaIS was conducted under a population-based open cohort design in Goto City, located in the remote islands of Nagasaki Prefecture, Japan (Figure 1). Conducting cohort studies in remote islands makes it easier to thoroughly follow participants and register their diseases. Additionally, we were granted permission to utilize various real-world data for this study, including our original drug dispensing data, with the support of Goto City. Our cohort study is distinctive in that it enables us to employ comprehensive disease registries and real-world data. This cohort study could serve as a reference and provide valuable insight into aging and risk factors associated with noncommunicable diseases and disabilities. Initially, we had planned for a 10-year study period and intended to extend it as necessary.

Researchers from various fields were involved in this study. The central office was the Nagasaki University Island Medical Research Institute in the Department of Island and Community Medicine, which had two full-time researchers endowed by Nagasaki Prefecture and Goto City, located at Nagasaki Goto Chuoh Hospital (Nagasaki Goto Central Hospital) in Goto, Nagasaki, Japan. Furthermore, the Nagasaki University Center for Advanced Preventive Medical Sciences, located at the Goto City Fukue General Health and Welfare Center, had two full-time researchers who collaborated in the management of this study. These institutes and several departments (Public Health, Immunology and Rheumatology, Oral Health, and Community Medicine) at Nagasaki University were organized into a steering committee to manage and control the progress of this study. The Department of General Medicine at Nagasaki University joined the Steering Committee in 2018. In addition, many departments, including the School of Pharmaceutical Sciences and the School of Health Sciences (Nursing, Physical Therapy, and Occupational Therapy), collaborated in this study as research members. We established an integrated database with sufficient security measures to store the collected cohort information and health checkup data.

The study protocol was developed by the research members and approved by the Ethics Committee of Nagasaki University Graduate School of Biomedical Sciences (project registration number: 14051404 [latest version: 16th edition]). The study was performed in accordance with the ethical standards of the 1964 Declaration of Helsinki and its later amendments.

## PARTICIPANTS

The target population in this study consisted of the 26,900 residents (12,008 men and 14,892 women) aged ≥40 years, based on the estimation by the National Institute of Population and Social Security Research in 2013.^15^ We performed our own health checkups, named “Arteriosclerosis Health Checkups,” and recruited study participants at these checkups. Prior to the novel coronavirus disease 2019 (COVID-19) pandemic, we distributed information leaflets in advance to eligible participants regarding these checkups. Residents who consented in writing to participate in this study were included.

Our own health checkups were an addition to the annual standardized health checkups for residents aged ≥40 years, which were conducted by the municipality according to the Act on Assurance of Medical Care for Elderly People and involved the assembly at a specific date and place of large numbers of residents mainly registered to receive National Health Insurance.^16^ After the COVID-19 pandemic, annual standardized health checkups were offered by appointment only, which reduced the number of participants receiving our checkups. In Japan, these standardized health checkups by the municipality were more frequently attended by women than men because men commonly underwent health checkups at their workplace or a comprehensive medical examination supported by their employer.^16^ Adults aged <40 years could also undergo these health checkups and participate in our study if they intended to.

**Figure 2.**
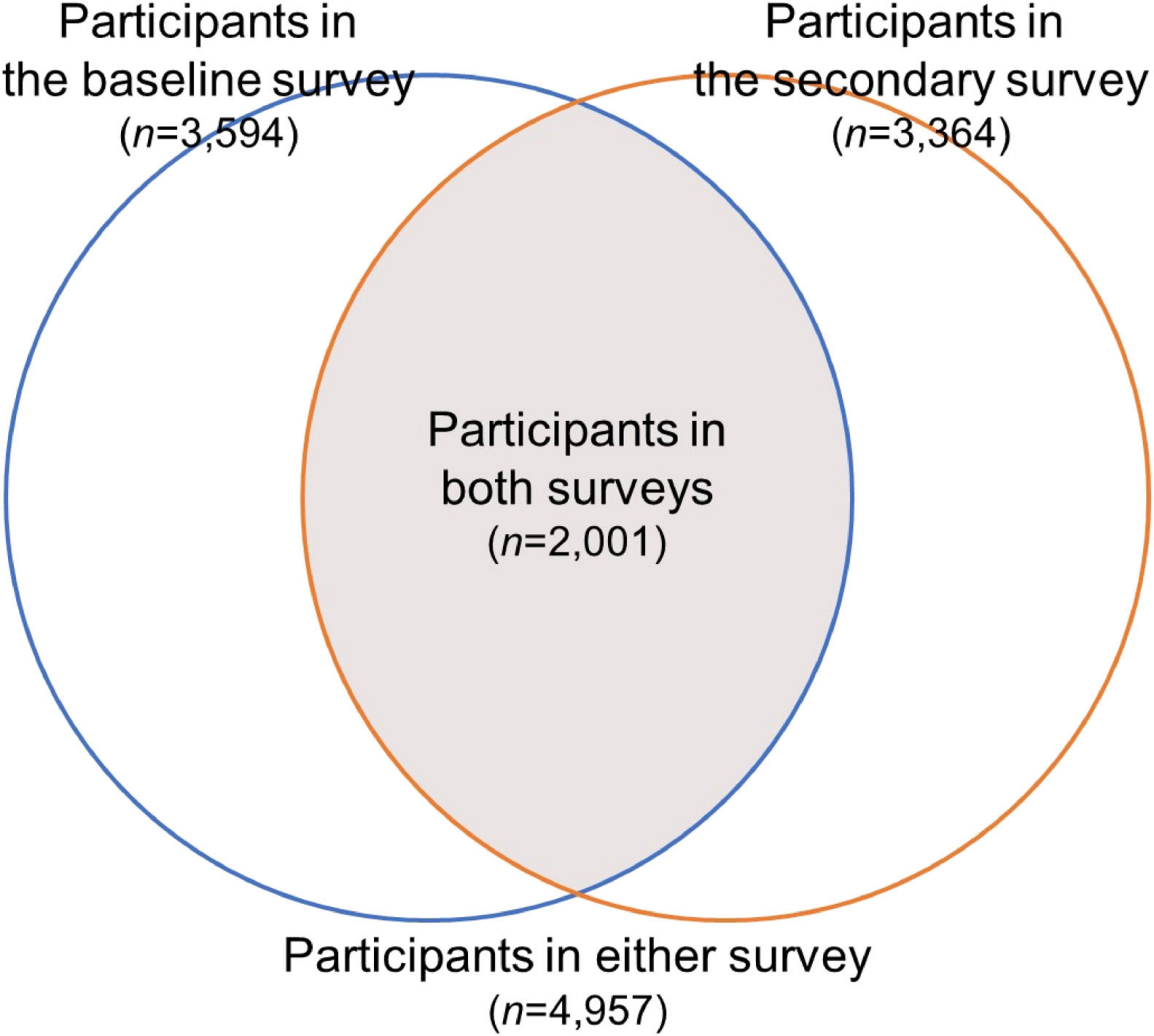
Venn diagram of participants in the baseline and secondary surveys of the Nagasaki Islands Study (NaIS)

As shown in Figure 2, a total of 4,957 residents participated in either the baseline or secondary surveys and were followed up; 3,594 and 3,364 residents (aged 27–96 and 28–98 years) participated in the baseline and secondary surveys, respectively. Regarding tertiary surveys, 325 and 571 residents underwent our health checkups in fiscal years (FYs) 2021 and 2022, respectively, and the survey will continue beyond 2023. The proportion of participants to residents who underwent standardized health checkups by the municipality was above 90% in all years.

## OUTCOMES AND FOLLOW-UP

We started to follow-up with the participants for vital status (death), migration, and occurrence of diseases. Information on the vital status and migration of the participants was obtained from the Goto City Municipal Office with permission each time. Out of 4,957 participants in either the baseline or secondary surveys, 4,564 (92.1%) were followed up, 242 (4.9%) died, and 151 (3.0%) dropped out owing to relocation as of October 31, 2021.

Regarding the occurrence of diseases, we extracted detailed information from the medical records into our specific registration forms at all hospitals in Goto City (Nagasaki Goto Chuoh [Central] Hospital, Nagasaki Tomie Hospital, Saint Mary Hospital, and Gunge Hospital), such as data on myocardial infarction, stroke, and fractures. To obtain disease incidence data, we initially categorized potential cases using the diagnosis from medical expenses and extracted detailed information, such as medical history, symptoms, and confirmatory tests, from the medical records into cohort-specific registration forms. The detailed information on the diagnosis of myocardial infarction included chest symptoms indicating myocardial ischemia, laboratory data on myocardial markers (such as troponin T), abnormal Q wave and ST-segment elevation in the electrocardiogram, and coronary angiography findings; those on stroke diagnosis included focal neurologic deficits, symptom duration (lasting ≥24 hours or less), and stroke imaging studies; and those on fracture diagnosis included fracture onset and imaging studies. Data on HTLV-1-associated uveitis was collected from the medical records of all hospitals and clinics that may treat eye diseases in Goto City (Nagasaki Goto Chuoh [Central] Hospital, Nagasaki Tomie Hospital, and Dake Eye Clinic), which were all diagnosed by certified ophthalmologists.

Additionally, we have been allowed to utilize real-world data, such as the Japanese National Health Insurance Database (Kokuho database), which provides comprehensive information on diagnostic codes and treatments in clinics and hospitals for beneficiaries (aged <75 years).^17^^,^^18^ We also have been permitted to use data from the Long-Term Care Insurance, which includes institutional and community-based care services to support daily life,^19^ and drug dispensing data collected from pharmacies in Goto City.^12^ Moreover, we are in the process of applying for permission to use data from the Latter-Stage Elderly Healthcare System, which covers all residents aged ≥75 years in Japan and provides comprehensive information similar to that of the Kokuho database.^17^

## MEASUREMENT

**Figure 3.**
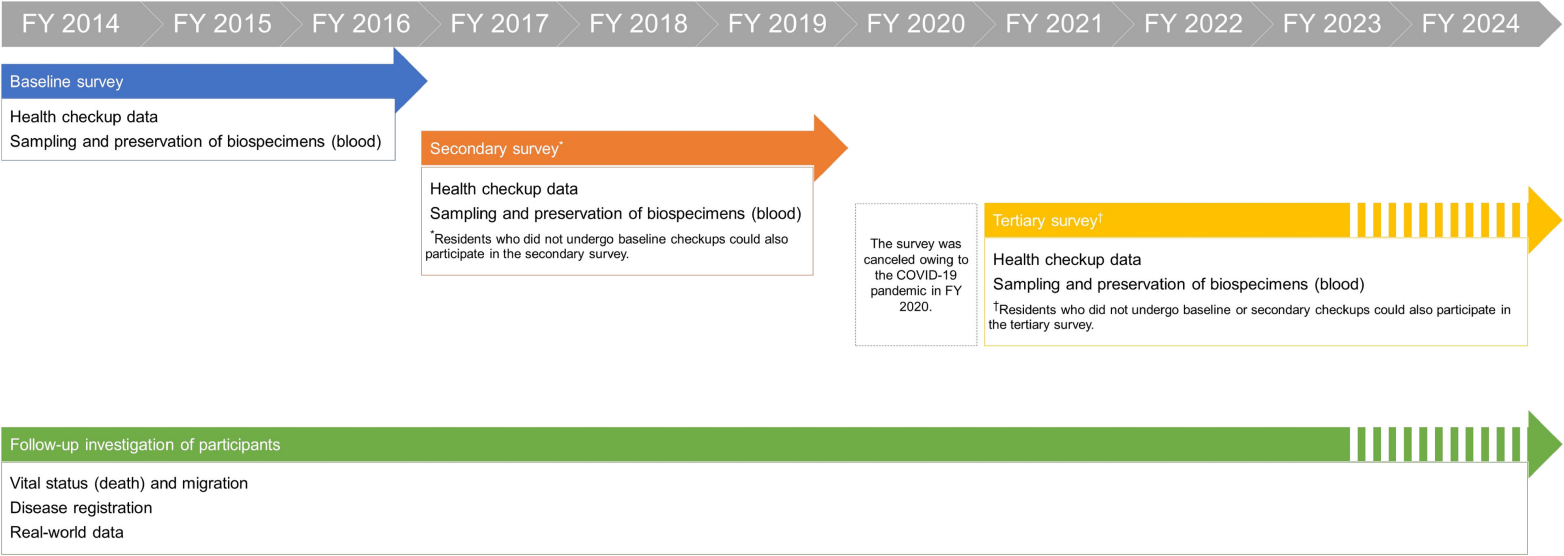
Study design showing the periods of baseline, secondary, and tertiary surveys of the Nagasaki Islands Study (NaIS). FY, fiscal year.

Before the COVID-19 pandemic, we had planned on performing our own health checkups as surveys across different areas of Goto City for over 3 years and conducted baseline surveys in Fukue in FY 2014, Tamanoura and Naru in FY 2015, and Tomie, Miiraku, Kishiku, and Hisaka in FY 2016 (Figure 1). We then performed the same checkups as secondary surveys during FYs 2017–2019. The health checkups were canceled in 2020 owing to the COVID-19 pandemic. Starting in 2021, we resumed conducting checkups as tertiary surveys. We conducted these surveys in Fukue during FYs 2021 and 2022 and in Tamanoura, Naru, and Kishiku in 2023. Residents who did not undergo baseline checkups could also participate in the later surveys. The study design is illustrated in Figure 3.

Our own checkups consisted of medical interviews, physical examinations, blood and urine tests, body composition measurements, osteoporosis screening, arterial stiffness measurements, and carotid ultrasonography. Some participants underwent additional dental examinations if desired, rather than all, because these examinations can take a bit of time to conduct.

The municipality conducted annual health checkups at several facilities throughout Goto City to facilitate residents’ accessibility. We visited almost all facilities in the target areas for baseline and secondary surveys and facilities with COVID-19 infection control for tertiary survey. We could not perform osteoporosis screening, arterial stiffness measurements, or dental examinations in some facilities because they were too small to accommodate. The components of our own health checkups are shown in Table 1.

**Table 1.** Components of the Nagasaki Islands Study (NaIS) health checkups

	Baseline survey (FY 2014–2016)			Secondary survey (FY 2017–2019)			Tertiary survey (since FY 2021)		
		
	FY 2014	FY 2015	FY 2016	FY 2017	FY 2018	FY 2019	FY 2021	FY 2022	FY 2023
Medical interview	All	All	All	All	All	All	All	All	All
Physical examination	All	All	All	All	All	All	All	All	All
Tongue pressure		All	All	All	All	All			All
Blood test	All	All	All	All	All	All	All	All	All
Bone-specific alkaline phosphatase		All	All	All	All	All	All	All	All
TRACP-5b and anti-HTLV-1 antibody			All	All	All	All	All	All	All
HsCRP				All	All	All			
CRP							All	All	All
EBV anti-EA IgG				All					
Urine test	All	All	All	All	All	All	All	All	All
Body composition measurement				All	All	All	All	All	All
Osteoporosis screening		Partial^a^	Partial^a^	Partial^a^	Partial^a^	Partial^a^	All	All	Partial^a^
Arterial stiffness measurement	Partial^a^	Partial^a^	Partial^a^	Partial^a^	Partial^a^	Partial^a^	All	All	Partial^a^
API and AVI	Partial^a^	Partial^a^	Partial^a^	Partial^a^	Partial^a^	Partial^a^			
Radial AI	Partial^a^	Partial^a^	Partial^a^	Partial^a^	Partial^a^				
Carotid ultrasonography	All	All	All	All	All	All	All	All	All
Dental examination	Partial^b^	Partial^b^	Partial^b^	Partial^b^	Partial^b^	Partial^b^	Partial^b^	Partial^b^	Partial^b^
Oral microbiota		Partial^b^	Partial^b^	Partial^b^	Partial^b^	Partial^b^			Partial^b^
Masticatory performance								Partial^b^	Partial^b^

“All” indicates that almost all participants underwent these examinations, and “partial” indicates that not all participants underwent them.

AI, augmentation index; API, arterial pressure-volume index; AVI, arterial velocity-pulse index; CRP, C-reactive protein; EBV anti-EA IgG, Epstein-Barr virus anti-early antigen immunoglobulin G; FY, fiscal year; hsCRP, high-sensitivity C-reactive protein; HTLV-1, human T-cell leukemia virus type 1; TRACP-5b, tartrate-resistant acid phosphatase 5b.

^a^We could not perform these examinations in facilities that were too small to accommodate.

^b^Only participants who wanted underwent these examinations because they can take a bit of time to conduct.

### Medical interview

During health checkups, trained interviewers asked participants about their medical history, smoking (including passive smoking), alcohol intake, exercise frequency, reproductive factors (menstruation, menopause, and delivery), sleep duration and disturbance, frailty, somatic symptoms (including rheumatoid arthritis symptoms), psychological distress score on the Kessler Psychological Distress Scale (K6),^20^^,^^21^ and living arrangements, according to the standardized questionnaire by the Japanese Ministry of Health, Labour and Welfare and our additional questionnaire.

### Physical examination

The participants’ height, body weight, waist circumference, blood pressure, and handgrip strength were measured. Tongue pressure was also measured since FY 2015 (the measurements were canceled due to the COVID-19 pandemic in 2021 and 2022). Height and body weight, with light clothing, were measured using an automatic body composition analyzer (BF-220; Tanita, Itabashi, Tokyo, Japan). Waist circumference was measured by touching a measuring tape on the participant’s abdomen in the horizontal plane at the level of the iliac crest. Blood pressure was measured using an automatic sphygmomanometer (HEM-907; Omron, Kyoto, Kyoto, Japan) placed on the arms of the participants in a sitting position. If the blood pressure level was excessively high or unreliable, the measurement was repeated or manual equipment was used. Handgrip strength was measured using a handgrip dynamometer (Smedley Dynamometer 0-1019-01; Matsumiya Ika Seiki Seisakujo, Edogawa, Tokyo, Japan) with adjustment such that the second proximal phalanxes of each participant were positioned around the handlebars and with an arm extended in a natural standing position. Handgrip strength was measured twice for each hand. The maximum voluntary tongue pressure (MVTP) against the palate, which has diagnostic value for dysphagia,^22^ was measured using a tongue pressure measurement device (JMS-TPM; JMS Co., Ltd., Hiroshima, Hiroshima, Japan). For measuring MVTP, the participants were placed in a relaxed sitting position and asked to compress the disposal balloon by raising their tongues using maximum voluntary effort.

### Blood and urine test

Blood and urine samples were collected from the participants with informed consent. The participants were assumed to have fasted, as they had been instructed to fast before undergoing a health checkup. At the baseline survey, we collected blood laboratory data such as complete blood count (including reticulocytes), levels of aspartate and alanine aminotransferase, alkaline phosphatase, gamma-glutamyl transferase, electrolytes (sodium, potassium, calcium, and phosphorus), creatinine, uric acid, lipids (triglycerides and low- and high-density lipoprotein cholesterol), glucose, hemoglobin A1c, and anti-cyclic citrullinated peptide antibody, which is a highly specific biomarker in rheumatoid arthritis.^23^ Moreover, we collected urine data based on dipstick urinalysis and sodium and creatinine levels. We additionally examined a bone formation marker (bone-specific alkaline phosphatase) since FY 2015, a bone resorption marker (tartrate-resistant acid phosphatase 5b), and anti-HTLV-1 antibody, based on chemiluminescent enzyme immunoassay since FY 2016. Since the secondary and tertiary surveys, we have additionally obtained blood laboratory data for an inflammatory marker (high-sensitivity C-reactive protein in the secondary survey, which was switched to C-reactive protein at the beginning of the tertiary survey). In FY 2017, we measured Epstein-Barr virus (EBV) anti-early antigen immunoglobulin G (IgG) as an indicator of EBV reactivation.

Laboratory tests were based on the standard laboratory procedures of SRL, Inc. (Shinjuku, Tokyo, Japan). Centrifugation was performed immediately after blood collection. All residual samples of serum and whole blood were stored at −80°C after being centrifuged for 10 minutes at 3,000 rpm in the case of serum. We extracted genomic deoxyribonucleic acid (DNA) from all collected whole blood samples and performed genomic analyses for such as human leukocyte antigen (HLA) genotypes.^24^ Sera were divided into several tubes for future multiplexed immunoassays using a fluorescence bead-based suspension array system, used to quantitatively measure micro-amount of proteins (cytokines, chemokines, and hormones).^25^

### Body composition measurement

Body composition was analyzed by multifrequency bioelectrical impedance analysis using an InBody 430 (InBody Japan, Koto, Tokyo, Japan) since the secondary survey.

### Osteoporosis screening

Since FY 2015, we measured heel quantitative ultrasound parameters (broadband ultrasound attenuation, speed of sound, and calcaneal stiffness index) through the participant’s right calcaneal bone using ultrasound bone densitometer (Achilles InSight; GE Lunar Corp., Madison, WI, USA). These parameters are alternatives to bone mineral density, which is the main predictive indicator of osteoporotic fracture.^26^

### Arterial stiffness measurement

We measured several indicators of arterial stiffness, including the cardio-ankle vascular index (CAVI), arterial pressure-volume index (API), arterial velocity-pulse index (AVI), and radial augmentation index (AI). The measurement of AI was stopped at the end of FY 2018, while those of API and AVI were stopped at the end of FY 2019. CAVI was an indicator of arterial stiffness not affected by blood pressure, unlike pulse wave velocity, and measured by a vascular screening system (VaSera VS-1000; Fukuda Denshi, Bunkyo, Tokyo, Japan) in a supine position.^27^ API and AVI were measured using a cuff-based oscillometric device (PASESA AVE-1500; Shisei Datum, Machida, Tokyo, Japan).^28^ The cuff was wrapped around one side of the upper arm of seated participants. Radial AI was defined as the ratio of the central to brachial pulse pressure, measured using an automated applanation tonometer (HEM-9000AI; Omron, Kyoto, Kyoto, Japan).^29^

### Carotid ultrasonography

We measured the carotid intima-media thickness (CIMT) as an indicator of atherosclerosis according to a previously described protocol.^30^ Experienced vascular examiners identified the edge of the first bright line (internal membrane; lumen-intima interface) and the second bright line (external membrane; media-adventitia interface) of the left and right common carotid arteries of the participants using a 10-MHz linear array ultrasound transducer (LOGIQ Book XP; GE Healthcare, Milwaukee, WI, USA) in Fowler’s position. The maximum values for the left- and right-sided CIMT, which were identified as the distance from the internal to the external membrane, were calculated using automated digital edge-detection software (IntimaScope; MediaCross, Ota, Tokyo, Japan), which can recognize the edges of the internal and external membranes of the artery semi-automatically and determine the distances at a sub-pixel level (estimated to be 0.01 mm).^31^

### Dental examination

Additional dental examinations were performed if the participants desired. The participants lay supine on a bed. Under illumination using a dental mirror and a periodontal probe, trained dentists assessed the oral health status, including the number of teeth, caries status, types of prosthetic restoration, probing pocket depth, bleeding on probing, clinical attachment level, debris index, calculus index, and wearing of removable dentures. Functional tooth units (FTUs) were calculated according to the number of opposing premolars or molar teeth by assigning one FTU to two opposing premolars and two FTUs to two opposing molars.^32^ Probing pocket depth was measured using a periodontal probe at the mesio-buccal and mid-buccal sites for all present teeth, excluding the third molars, according to the method of the Third National Health and Nutrition Examination Survey in the United States.^33^ We also collected data on toothbrushing habits and oral health-related quality of life.^34^^,^^35^

Moreover, we analyzed the oral microbiota since FY 2015 (the measurements were canceled owing to the COVID-19 pandemic in 2021 and 2022). We detected bacteria causing periodontal disease from participants’ chewing-stimulated whole saliva samples by polymerase chain reaction (PCR) amplification and sequencing of V1 and V2 regions of 16S ribosomal ribonucleic acid (rRNA) gene. This was done using a collection and stabilization kit (OMNIgene^®^-ORAL OM-501; DNA Genotek, Ottawa, ON, Canada), a simultaneous purification kit (QIAamp MinElute Virus Spin Kit; QIAGEN, Venlo, Limburg, Netherlands), and a next-generation sequencing system (MiSeq; Illumina, Inc., San Diego, CA, USA).^36^

Since 2022, we have evaluated masticatory performance by measuring the glucose concentration obtained from a chewed gummy jelly (Glucorumm^®^; GC Co. Ltd, Bunkyo, Tokyo, Japan) according to a previously described protocol.^37^^,^^38^ We instructed participants to chew gummy jelly on their habitual chewing side for 20 seconds, hold 10 mL of water in their mouth for a moment, and then spit it into a cup. After filtering out the gummy jelly from the spat water in the cup, we collected the filtrate, including saliva, and measured the glucose concentration using a glucose testing device (Gluco Sensor GS-II^®^; GC Co. Ltd, Bunkyo, Tokyo, Japan).

## BASELINE CHARACTERISTICS

The total number of participants in the baseline and secondary surveys is presented in Figure 2, and those in each area are shown in Table 2. Overall, 4,957 residents participated in either the baseline or secondary surveys and were followed up. In the baseline and secondary surveys, 3,594 and 3,364 residents (aged 27–96 and 28–98 years, respectively) consented to participate (some participants took each survey more than once). Excluding participants aged <40 years, of these 3,530 (12.6% of 28,016, total population aged ≥40 years in 2014) and 3,304 (12.1% of 27,235, in 2017) individuals participated in the study. A total of 2,001 residents participated in both the baseline and secondary surveys. Regarding tertiary surveys, 325 and 571 residents underwent our health checkups in FYs 2021 and 2022, respectively, and the survey will continue beyond 2023.

**Table 2.** Number of total participants in the baseline and secondary surveys of the Nagasaki Islands Study (NaIS), classified by area in Goto City

Area	Baseline survey					Secondary survey					Total	
		
	Year	Total participants in NaIS (Aged <40 years in the fiscal year)		Total population of Goto City aged ≥40 years in 2014^a^		Year	Total participants in NaIS (Aged <40 years in the fiscal year)		Total population of Goto City aged ≥40 years in 2017^a^		Participants in both baseline and secondary surveys^b^ in NaIS (Aged <40 years in the fiscal year^b,c^)	
				
		Women	Men	Women	Men		Women	Men	Women	Men	Women	Men
Fukue	2014	1,047 (23)	555 (15)	8,787	7,101	2017	1,110 (19)	613 (12)	8,713	7,118	583 (8)	306 (7)
Tamanoura	2015	210 (0)	122 (0)	666	566	2018	178 (3)	113 (7)	613	525	136 (0)	76 (0)
Naru	2015	195 (7)	128 (1)	1,142	1,008	2018	152 (4)	91 (2)	1,035	946	99 (1)	66 (1)
Miiraku	2016	211 (6)	146 (1)	1,156	986	2019	175 (0)	128 (2)	1,079	958	105 (0)	72 (0)
Kishiku	2016	279 (5)	204 (2)	1,408	1,156	2019	246 (5)	160 (2)	1,345	1,100	175 (1)	110 (0)
Tomie	2016	248 (5)	191 (2)	2,014	1,705	2019	222 (4)	144 (4)	1,906	1,618	132 (1)	94 (1)
Hisaka	2016	44 (1)	40 (2)	166	155	2019	38 (1)	29 (0)	144	135	27 (0)	20 (0)

Duplicates		24 (6)	2 (0)				26 (4)	9 (1)				

Total		2,210 (41)	1,384 (23)	15,339	12,677		2,095 (32)	1,269 (28)	14,835	12,400	1,257 (11)	744 (9)

Residents in one area may have participated in the survey in another area, and some participants may have completed each survey more than once, leading to duplicates.

^a^As of September 30, 2014, or 2017, respectively.

^b^The participants who underwent surveys across different areas were classified according to the area in which they first underwent the baseline survey.

^c^At the baseline survey.

The number of participants who underwent additional dental examinations in each area is shown in Table 3. Some participants did not undergo dental examinations at their own request or because the facilities were too small to accommodate them. No participant exclusively underwent dental checkups. Overall, 2,164 residents underwent dental examinations in either the baseline or secondary survey. In the baseline and secondary surveys, 1,338 and 1,363 residents, respectively, consented to participate. Excluding participants aged <40 years, of these 1,307 (4.7% of the total population aged ≥40 years in 2014) and 1,325 (4.9% in 2017) residents participated. A total of 537 residents participated in both the baseline and secondary surveys. Regarding tertiary surveys, 159 and 421 residents underwent dental examinations in FYs 2021 and 2022, respectively, and the survey will continue beyond 2023.

**Table 3.** Number of participants undergoing dental examinations in the baseline and secondary surveys of the Nagasaki Islands Study (NaIS), classified by area in Goto City

Area	Baseline survey					Secondary survey					Total	
		
	Year	Participants undergoing dental examination in NaIS (Aged <40 years in the fiscal year)		Total population of Goto City aged ≥40 years in 2014^a^		Year	Participants undergoing dental examination in NaIS (Aged <40 years in the fiscal year)		Total population of Goto City aged ≥40 years in 2017^a^		Participants undergoing dental examination in both baseline and secondary surveys^b^ in NaIS (Aged <40 years in the fiscal year^b,c^)	
				
		Women	Men	Women	Men		Women	Men	Women	Men	Women	Men
Fukue	2014	373 (9)	224 (9)	8,787	7,101	2017	470 (13)	267 (8)	8,713	7,118	150 (2)	98 (4)
Tamanoura	2015	37 (0)	26 (0)	666	566	2018	39 (3)	36 (4)	613	525	11 (0)	13 (0)
Naru	2015	122 (5)	98 (1)	1,142	1,008	2018	68 (2)	60 (1)	1,035	946	38 (2)	38 (1)
Miiraku	2016	76 (3)	56 (1)	1,156	986	2019	74 (0)	52 (2)	1,079	958	34 (0)	22 (0)
Kishiku	2016	95 (0)	59 (0)	1,408	1,156	2019	93 (2)	53 (0)	1,345	1,100	50 (0)	29 (0)
Tomie	2016	89 (4)	58 (0)	2,014	1,705	2019	77 (1)	51 (2)	1,906	1,618	21 (1)	21 (0)
Hisaka	2016	19 (1)	14 (1)	166	155	2019	13 (1)	19 (0)	144	135	5 (0)	7 (0)

Duplicates		7 (3)	1 (0)				3 (1)	6 (0)				

Total		804 (19)	534 (12)	15,339	12,677		831 (21)	532 (17)	14,835	12,400	309 (5)	228 (5)

Residents in one area may have participated in the survey in another area, and some participants may have completed each survey more than once, leading to duplicates.

^a^As of September 30, 2014, or 2017, respectively.

^b^The participants who underwent surveys across different areas were classified according to the area in which they first underwent the baseline survey.

^c^At the baseline survey.

The proportion of men was 38.5% at the baseline and 37.7% in the secondary survey. The proportion of participants in the total population, except for those aged <40 years, was higher for women (14.4% at baseline and 14.1% in the secondary survey) than that for men (10.9% at baseline and 10.2% in the secondary survey).

The characteristics of the study participants are shown in Table 4. The mean age was 69.0 (standard deviation [SD], 11.2) years at baseline and 69.9 (SD, 11.3) years at secondary survey. The proportion of current smokers and habitual drinkers was higher among men than among women. The proportion of past smokers was relatively higher in our study than in the data from the National Health and Nutrition Survey (the proportion of past smokers was 3.3% among women aged ≥40 years and 15.7% among men aged ≥40 years in 2014).^39^^,^^40^ The higher proportion of past smokers might reflect the high production of leaf tobacco in Nagasaki Prefecture,^41^ which includes Goto City.

**Table 4.** Characteristics of participants in the baseline and secondary surveys of the Nagasaki Islands Study (NaIS)

	Baseline survey		Secondary survey	
	
	Women (*n* = 2,234)^a^	Men (*n* = 1,386)^a^	Women (*n* = 2,121)^a^	Men (*n* = 1,278)^a^
Age				
<39 (%)	2.1	1.7	1.7	2.3
40–49 (%)	4.2	4.3	3.9	4.8
50–59 (%)	10.5	9.7	8.5	8.0
60–69 (%)	33.3	34.3	30.7	29.0
70–79 (%)	33.4	32.5	35.2	35.3
80–89 (%)	15.7	16.6	19.0	19.2
≥90 (%)	0.8	1.0	0.9	1.5
Mean (SD)	68.8 (11.2)	69.2 (11.3)	69.9 (11.1)	69.8 (11.7)
Smoking status				
Never (%)	91.8	27.3	91.8	26.1
Past (%)	5.3	52.6	5.4	53.4
Current (%)	2.8	20.1	2.8	20.6
Missing (*n*)	4	3	0	0
Alcohol intake				
Never (%)	78.1	29.1	78.8	31.1
Past (%)	3.2	10.8	1.7	8.1
Sometimes (%)	14.8	24.3	15.7	21.8
Everyday (%)	4.0	35.8	3.9	39.0
Missing (*n*)	1	0	0	0
Height (cm)				
Mean (SD)	150.9 (6.4)	163.4 (6.6)	151.4 (6.2)	164.0 (6.5)
Weight (kg)				
Mean (SD)	52.0 (8.8)	63.1 (9.7)	51.9 (8.9)	63.8 (10.3)

SD, standard deviation.

^a^Includes duplicates who completed each survey more than once.

**Figure 4.**
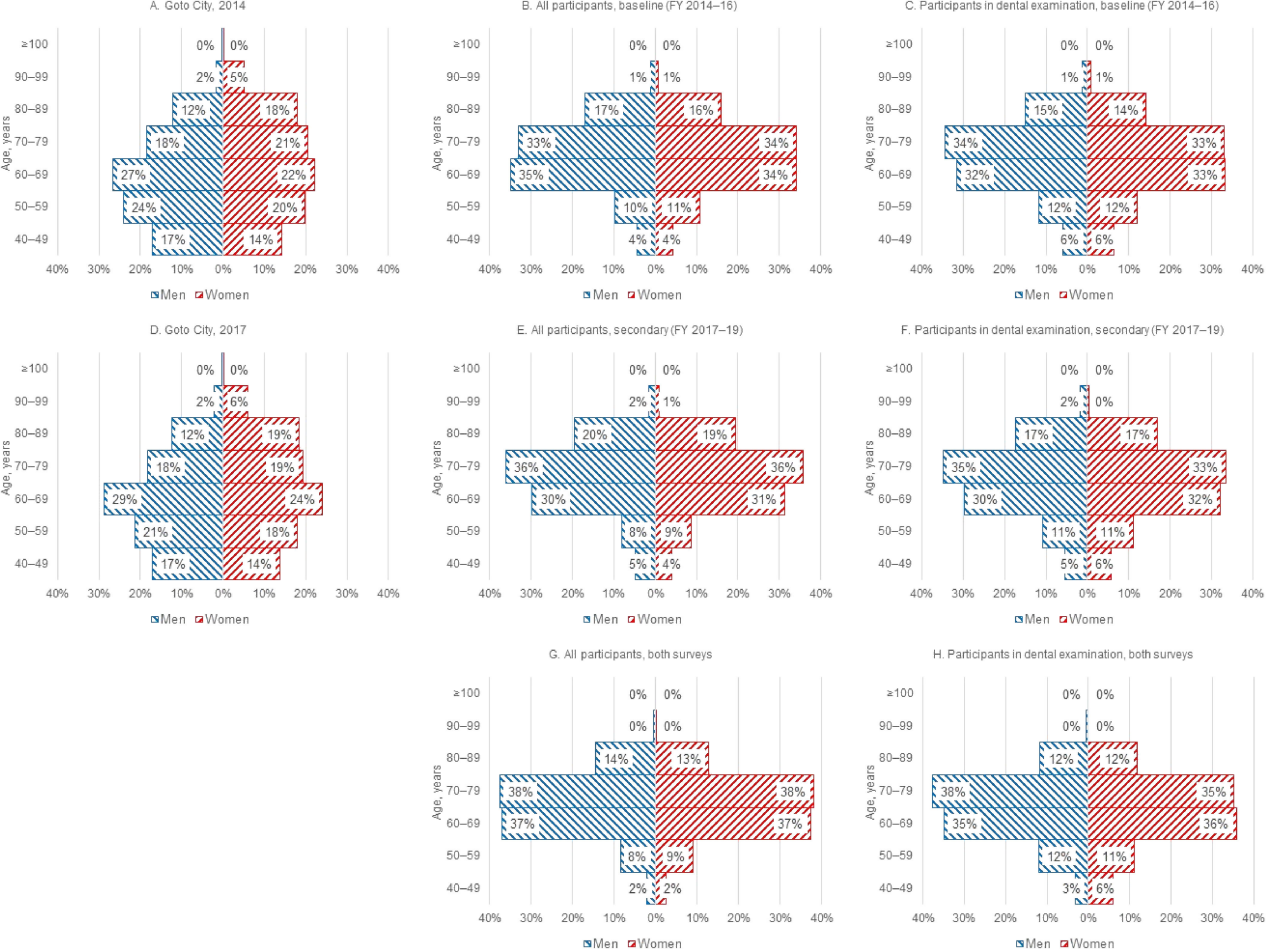
Age distribution of Goto City and participants in the baseline and secondary surveys of the Nagasaki Islands Study (NaIS). FY, fiscal year.

The age distribution of the participants older than 40 years in our study at baseline and the secondary survey is shown in Figure 4. Compared with the age distribution in Goto City (Figure 4A and Figure 4D), the distribution of the participants in our study was skewed toward the age groups of 60–69 and 70–79 years.

## STRENGTHS AND LIMITATIONS

We conducted a population-based open cohort study, mainly including middle-aged and older residents on remote islands in Japan. Researchers from various fields were involved in this study. This study was designed to identify the influence of aging on health conditions; elucidate risk factors for cardiovascular diseases, rheumatic and autoimmune diseases, dental diseases, frailty, sarcopenia, osteoporosis, and fracture; and contribute to their prevention. Conducting a cohort study in remote islands made it easier to thoroughly follow participants and register their diseases. Additionally, our cohort study is distinctive in that it allows us to utilize various real-world data as well as exhaustive disease registries with support from Goto City.

The issue of loss to follow-up, which might have influenced the internal validity, needs to be addressed. As of October 31, 2021, 3% of the participants in either the baseline or secondary surveys dropped out due to relocation. The low dropout rate is one of the strengths of our cohort study conducted in remote islands; meanwhile, the risk of bias owing to the loss to follow-up cannot be ignored as a limitation if these participants experienced severe diseases and disabilities leading them to move out of remote islands, which had few medical and nursing facilities.^42^

Additionally, this study had some limitations that affected the external validity. Our inclusion criteria were residents who underwent our own health checkups, which caused an issue of regional representativeness in terms of age and sex distribution. The proportion of participants aged 40–59 years was lower than expected based on the age distribution in Goto City (Figure 4). In addition, the proportion of men was lower than that of women. The reason might be that some residents aged 40–59 years, especially men, underwent health checkups at their workplace or underwent a comprehensive medical examination supported by their employer.^16^ The proportion of participants aged ≥80 years was also lower as some of them might need care or support for their daily living according to their physical and mental disability, resulting in difficulty in attending our health checkups. Moreover, those residents who attended health checkups would tend to be healthier as well as health conscious, and the Hawthorne effect, in which the mere awareness of being under observation might improve or modify their behavior, also affected external validity. Hence, the results of our study should be interpreted with caution.

## CONCLUSION

In conclusion, the NaIS has been undertaken to reveal the influence of aging and risk factors of noncommunicable diseases and disabilities, and to contribute towards better healthcare in the future. We were permitted to utilize exhaustive disease registries and various real-world data. This study is expected to provide evidence for improving the diagnosis and treatment of disorders that affect aging societies.

## References

[1] GBD 2013 DALYs and HALE Collaborators. Global, regional, and national disability-adjusted life years (DALYs) for 306 diseases and injuries and healthy life expectancy (HALE) for 188 countries, 1990–2013: quantifying the epidemiological transition. Lancet. 2015;386(10009):2145–2191. 10.1016/S0140-6736(15)61340-X 26321261 PMC4673910

[2] Yarnall AJ, Sayer AA, Clegg A, Rockwood K, Parker S, Hindle JV. New horizons in multimorbidity in older adults. Age Ageing. 2017;46(6):882–888. 10.1093/ageing/afx150 28985248 PMC5860018

[3] Global Burden of Disease Study 2013 Collaborators. Global, regional, and national incidence, prevalence, and years lived with disability for 301 acute and chronic diseases and injuries in 188 countries, 1990–2013: a systematic analysis for the Global Burden of Disease Study 2013. Lancet. 2015;386(9995):743–800. 10.1016/S0140-6736(15)60692-4 26063472 PMC4561509

[4] Cabinet Office, Government of Japan. Current situation and issues on remote populated territorial island areas [Tokutei yujin kokkyo rito chiiki no genjo to kadai]. https://www8.cao.go.jp/ocean/kokkyouritou/yuushiki/bunkakai/h28_01bunkakai/pdf/shiryou_05.pdf; 2016 Accessed 9,7,2023. Article in Japanese.

[5] Public Relations Office, Government of Japan. Life innovation: a society of health and longevity. Highlighting Japan. 2010;4(2):22–23.

[6] Palombo C, Kozakova M. Arterial stiffness, atherosclerosis and cardiovascular risk: pathophysiologic mechanisms and emerging clinical indications. Vascul Pharmacol. 2016;77:1–7. 10.1016/j.vph.2015.11.083 26643779

[7] England BR, Thiele GM, Anderson DR, Mikuls TR. Increased cardiovascular risk in rheumatoid arthritis: mechanisms and implications. BMJ. 2018;361:k1036. 10.1136/bmj.k1036 29685876 PMC6889899

[8] Melissaropoulos K, Bogdanos D, Dimitroulas T, Sakkas LI, Kitas GD, Daoussis D. Primary Sjögren’s syndrome and cardiovascular disease. Curr Vasc Pharmacol. 2020;18(5):447–454. 10.2174/1570161118666200129125320 31995009

[9] Kostopoulou M, Nikolopoulos D, Parodis I, Bertsias G. Cardiovascular disease in systemic lupus erythematosus: recent data on epidemiology, risk factors and prevention. Curr Vasc Pharmacol. 2020;18(6):549–565. 10.2174/1570161118666191227101636 31880245

[10] Sanz M, Marco Del Castillo A, Jepsen S, . Periodontitis and cardiovascular diseases: consensus report. J Clin Periodontol. 2020;47(3):268–288. 10.1111/jcpe.13189 32011025 PMC7027895

[11] Iwasaki T, Fukuda H, Hayashida H, . Status and factors of tooth loss on remote islands with no dentist. J Dent Health. 2016;66:445–451. Article in Japanese. 10.5834/jdh.66.5_445

[12] Maeda T, Nakamura H, Sugawara M. Establishment of medical corporation system using ICT and practice of big-data utilization. Jpn J Acute Med. 2018;42(8):955–962. Article in Japanese.

[13] Gessain A, Cassar O. Epidemiological aspects and world distribution of HTLV-1 infection. Front Microbiol. 2012;3:388. 10.3389/fmicb.2012.00388 23162541 PMC3498738

[14] Schierhout G, McGregor S, Gessain A, Einsiedel L, Martinello M, Kaldor J. Association between HTLV-1 infection and adverse health outcomes: a systematic review and meta-analysis of epidemiological studies. Lancet Infect Dis. 2020;20(1):133–143. 10.1016/S1473-3099(19)30402-5 31648940

[15] National Institute of Population and Social Security Research. Regional population projections for Japan: 2010–2040 (March 2013). https://www.ipss.go.jp/pp-shicyoson/e/shicyoson13/t-page.asp; 2013 Accessed 9,7,2023.

[16] Ikeda N, Saito E, Kondo N, . What has made the population of Japan healthy? Lancet. 2011;378(9796):1094–1105. 10.1016/S0140-6736(11)61055-6 21885105

[17] Ikegami N, Yoo BK, Hashimoto H, . Japanese universal health coverage: evolution, achievements, and challenges. Lancet. 2011;378(9796):1106–1115. 10.1016/S0140-6736(11)60828-3 21885107

[18] Nagai K, Iseki C, Iseki K, . Higher medical costs for CKD patients with a rapid decline in eGFR: a cohort study from the Japanese general population. PLoS One. 2019;14(5):e0216432. 10.1371/journal.pone.0216432 31100069 PMC6524806

[19] Tsutsui T, Muramatsu N. Japan’s universal long-term care system reform of 2005: containing costs and realizing a vision. J Am Geriatr Soc. 2007;55(9):1458–1463. 10.1111/j.1532-5415.2007.01281.x 17767690

[20] Kessler RC, Andrews G, Colpe LJ, . Short screening scales to monitor population prevalences and trends in non-specific psychological distress. Psychol Med. 2002;32(6):959–976. 10.1017/S0033291702006074 12214795

[21] Furukawa TA, Kawakami N, Saitoh M, . The performance of the Japanese version of the K6 and K10 in the World Mental Health Survey Japan. Int J Methods Psychiatr Res. 2008;17(3):152–158. 10.1002/mpr.257 18763695 PMC6878390

[22] Yoshida M, Kikutani T, Tsuga K, Utanohara Y, Hayashi R, Akagawa Y. Decreased tongue pressure reflects symptom of dysphagia. Dysphagia. 2006;21(1):61–65. 10.1007/s00455-005-9011-6 16544085

[23] Whiting PF, Smidt N, Sterne JA, . Systematic review: accuracy of anti-citrullinated peptide antibodies for diagnosing rheumatoid arthritis. Ann Intern Med. 2010;152(7):456–464; W155–W166. 10.7326/0003-4819-152-7-201004060-00010 20368651

[24] Kawaguchi S, Higasa K, Shimizu M, Yamada R, Matsuda F. HLA-HD: an accurate HLA typing algorithm for next-generation sequencing data. Hum Mutat. 2017;38(7):788–797. 10.1002/humu.23230 28419628

[25] Graham H, Chandler DJ, Dunbar SA. The genesis and evolution of bead-based multiplexing. Methods. 2019;158:2–11. 10.1016/j.ymeth.2019.01.007 30659874

[26] Arima K, Mizukami S, Nishimura T, . Epidemiology of the association between serum 25-hydroxyvitamin D levels and musculoskeletal conditions among elderly individuals: a literature review. J Physiol Anthropol. 2020;39(1):38. 10.1186/s40101-020-00249-3 33243295 PMC7690203

[27] Yambe T, Yoshizawa M, Saijo Y, . Brachio-ankle pulse wave velocity and cardio-ankle vascular index (CAVI). Biomed Pharmacother. 2004;58(Suppl 1):S95–S98. 10.1016/S0753-3322(04)80015-5 15754845

[28] Komine H, Asai Y, Yokoi T, Yoshizawa M. Non-invasive assessment of arterial stiffness using oscillometric blood pressure measurement. Biomed Eng Online. 2012;11:6. 10.1186/1475-925X-11-6 22325084 PMC3359259

[29] Kohara K, Tabara Y, Oshiumi A, Miyawaki Y, Kobayashi T, Miki T. Radial augmentation index: a useful and easily obtainable parameter for vascular aging. Am J Hypertens. 2005;18(S1):11S–14S. 10.1016/j.amjhyper.2004.10.010 15683726

[30] Hara T, Takamura N, Akashi S, . Evaluation of clinical markers of atherosclerosis in young and elderly Japanese adults. Clin Chem Lab Med. 2006;44(7):824–829. 10.1515/CCLM.2006.149 16776627

[31] Yanase T, Nasu S, Mukuta Y, . Evaluation of a new carotid intima-media thickness measurement by B-mode ultrasonography using an innovative measurement software, intimascope. Am J Hypertens. 2006;19(12):1206–1212. 10.1016/j.amjhyper.2006.05.010 17161764

[32] Hildebrandt GH, Dominguez BL, Schork MA, Loesche WJ. Functional units, chewing, swallowing, and food avoidance among the elderly. J Prosthet Dent. 1997;77(6):588–595. 10.1016/S0022-3913(97)70100-8 9185051

[33] Brown LJ, Brunelle JA, Kingman A. Periodontal status in the United States, 1988–1991: prevalence, extent, and demographic variation. J Dent Res. 1996;75(2S):672–683. 10.1177/002203459607502S07 8594091

[34] Atchison KA, Dolan TA. Development of the Geriatric Oral Health Assessment Index. J Dent Educ. 1990;54(11):680–687. 10.1002/j.0022-0337.1990.54.11.tb02481.x 2229624

[35] Naito M, Suzukamo Y, Nakayama T, Hamajima N, Fukuhara S. Linguistic adaptation and validation of the General Oral Health Assessment Index (GOHAI) in an elderly Japanese population. J Public Health Dent. 2006;66(4):273–275. 10.1111/j.1752-7325.2006.tb04081.x 17225823

[36] Yano Y, Hua X, Wan Y, . Comparison of oral microbiota collected using multiple methods and recommendations for new epidemiologic studies. mSystems. 2020;5(4):e00156-20. 10.1128/mSystems.00156-20 32636335 PMC7343307

[37] Shiga H, Kobayashi Y, Katsuyama H, Yokoyama M, Arakawa I. Gender difference in masticatory performance in dentate adults. J Prosthodont Res. 2012;56(3):166–169. 10.1016/j.jpor.2012.02.001 22613955

[38] Imamura Y, Chebib N, Ohta M, . Masticatory performance in oral function assessment: alternative methods. J Oral Rehabil. 2023;50(5):383–391. 10.1111/joor.13421 36691751

[39] National Institute of Health and Nutrition. Section of the National Health and Nutrition Survey. https://www.nibiohn.go.jp/eiken/english/research/project_nhns.html; 2018 Accessed 9,7,2023.

[40] Ministry of Health, Labour and Welfare. National Health and Nutrition Survey [Kokumin kenko, eiyo chosa]. https://www.mhlw.go.jp/toukei/itiran/gaiyo/k-eisei.html; 2022 Accessed 9,7,2023. Article in Japanese.

[41] Japan Tobacco Growers Association. About tobacco cultivation [Tabako kosaku ni tsuite]. http://www.jtga.or.jp/outline/; 2022 Accessed 9,7,2023. Article in Japanese.

[42] Maeda T, Nakazato M, Seo M, . Medical care for the elderly in rural areas. Nihon Ronen Igakkai Zasshi. 2007;44(1):58–61. Article in Japanese. 10.3143/geriatrics.44.58 17337852

